# Cardiac magnetic resonance quantified epicardial fat volume is associated with complex coronary artery disease among diabetics

**DOI:** 10.1186/s12933-025-02606-x

**Published:** 2025-02-07

**Authors:** Shimaa Sayed Khidr, Bakeer Mohamed Bakeer, Hatem Abdel-Rahman Helmy, Heba Mahmoud El-Naggar

**Affiliations:** Department of Cardiovascular Medicine, Assiut University Heart Hospital, Assiut, 71526 Egypt

**Keywords:** Epicardial fat volume, Pericardial fat volume, Cardiac magnetic resonance, Syntax score, Diabetes mellitus

## Abstract

**Background:**

Epicardial and pericardial adipose tissues are two distinct types of visceral fat in close adherence to the heart and were found to be increased among diabetics.

**Aim:**

To investigate the correlation between cardiac magnetic resonance (CMR)-quantified epicardial (EFV) and pericardial fat (PFV) volumes and the complexity of coronary artery disease (CAD) among diabetic and non-diabetic patients.

**Methods:**

This was a cross-sectional study that included 111 patients having CAD as indicated by coronary angiography and who underwent CMR. Epicardial and pericardial fat volumes were measured along short-axis CMR-derived images. CAD severity and complexity were evaluated using the syntax score (SS). Patients were classified into diabetic and non-diabetic groups based on their HbA1c and were compared regarding clinical, angiographic, and CMR data. Those with high SS were compared against low/intermediate SS. The correlation of measured EFV and PFV with the SS was evaluated, and possible predictors for high-SS were assessed.

**Results:**

Diabetic patients (*n* = 64, 57.7%) had significantly high syntax scores, and significantly larger absolute and indexed EFV and PFV compared to non-diabetics. Both EFV and PFV showed a significant positive correlation with HbA1c and SS. EFV ≥ 119.55 ml significantly predicted high-SS (AUC = 0.84, 95%CI = 0.76–0.91, sensitivity = 77% and specificity = 82.5%) among the study population. Different cutoff points of EFV significantly predicted high SS among diabetics and non-diabetics with respective reasonable sensitivity and specificity. Age and EFV were consistently predictive of high SS on different multivariable regression models.

**Conclusion:**

Increased epicardial adipose tissue was a significant independent predictor of severe and complex CAD, representing a possible risk marker and potential therapeutic target, particularly among diabetics.

**Supplementary Information:**

The online version contains supplementary material available at 10.1186/s12933-025-02606-x.

## Introduction

Epicardial and pericardial adipose tissues are two distinct types of visceral fat in close adherence to the heart and coronaries and were found to be increased among diabetics. A mutual relation between increased epicardial adipose tissue (EAT) and insulin resistance as one of the proposed mechanisms of diabetes has been suggested, whereby a vicious circle of increase in one would impact the other [1].

It has been demonstrated that in patients with diabetes, epicardial fat volume is significantly increased and becomes remodeled into a more proinflammatory phenotype with consequently deleterious effects, increasing the incidence and severity of CAD as well as the risk of adverse cardiac events and mortality [2]. On the other hand, abnormally increased EAT leads to excess secretion of bioactive substances with subsequent systemic inflammation, altered plasma cholesterol levels, and insulin resistance leading to DM and enhanced atherosclerosis [3, 4].

Cardiac magnetic resonance (CMR) imaging would precisely assess both epicardial and pericardial fat volumes, given its high soft tissue characterization with signal-to-noise ratio differentiating fat from surrounding tissues, as well as its ability to follow fat invagination and extensions [5].

This study sought to comprehensively assess both epicardial (EFV) and pericardial (PFV) fat volumes using CMR among diabetics compared to non-diabetics and to evaluate their association with the severity and complexity of CAD assessed by the syntax score.

## Methods

This was a cross-sectional observational study from March 2021 to December 2023 that included patients having CAD as indicated on coronary angiogram and who presented to our institutional CMR unit for viability assessment. Patients with contraindications for CMR (MRI non-conditional devices, claustrophobia, and estimated glomerular filtration rate < 30 ml/min/1.73m^2^, noting that gadolinium was not required for EFV measurement but for the viability study), and those who had a time-interval between the coronary angiography and the CMR-study exceeding three months were excluded.

The presence of CAD risk factors (smoking, hypertension, dyslipidemia) was assessed and anthropometric measurements of weight, height, body mass index (BMI), and body surface area (BSA) were recorded. Laboratory data included an assessment of total cholesterol, low-density lipoprotein (LDL), high-density lipoprotein (HDL), and triglyceride levels. The atherogenic index of plasma (AIP) was calculated as the logarithm of the ratio of triglycerides to HDL [6]. Assessment of glycated hemoglobin (HbA1c) level was performed at the time of the CMR study. Based on the HbA1c cutoff for diagnosis of DM (≥ 6.5%), patients were classified into diabetic and non-diabetic groups. Diabetic patients were further classified into tight-control group (HbA1c 6.5–6.9%), relax-control group (HbA1c 7.0-8.4%), and uncontrolled group (HbA1c ≥ 8.5%) [7].

The coronary angiograms were evaluated by an experienced cardiologist with more than five years of experience, blinded to patients´ clinical and CMR data. The syntax score (SS) was used to objectively quantify CAD severity and complexity, calculated for each coronary lesion causing ≥ 50% luminal obstruction in vessels with a diameter of ≥ 1.5 mm [8]. Patients were divided according to 2-year rates of major adverse coronary events into three tertiles: low-SS (≤ 22), intermediate-SS (> 22–32), and high-SS (> 32) [9].

CMR was performed using a 1.5-Tesla scanner (Philips Ingenia Release 4.1.3.0, Philips Medical Systems, the Netherlands), using a phased array cardiac receiver coil. Standard cine steady-state free precession (SSFP) images of the left (LV) and right (RV) ventricles were acquired in the horizontal and vertical long-axis views and left ventricular outflow tract view, and a stack of short-axis images for volumetric and functional assessment was taken (TR/TE:3.1ms/1.5ms, flip angle:70°, FOV:300 mm, Voxel size:1.97/2.05/8.00 mm, 8 mm slice thickness with no gaps for short-axis images). Late gadolinium enhanced (LGE) images were acquired by phase-sensitive inversion recovery technique in 2-, 3-, and 4-axis views, together with 3–5 short-axis levels. Image analysis was performed offline using dedicated software (MR-Workspace R2.6.3.1). On cine images, the endocardial and epicardial contours were traced on end-diastolic and end-systolic frames to calculate corresponding LV and RV volumes. Maximum left atrial (LA) volume was measured using the biplane area-length method. For viability assessment, the LV 17-segment model was used to describe the enhancement distribution and extent [10].

Cardiac adipose tissue volumes were quantified on the SSFP short-axis slices (from the mitral annular level down to the last apical slice) in the end-diastolic phase by a single observer blinded to corresponding clinical and angiographic data. Epicardial fat was defined as the adipose tissue accumulated between the visceral pericardium and the myocardium [11]. Pericardial fat was defined as the fat located on the outer surface of the fibrous pericardium [12]. Accordingly, corresponding epicardial and pericardial fat areas subtended by manual tracing (using endo-contour) were measured at consecutive short-axis slices with integration over the horizontal and vertical long-axis images. The fat area measured in each slice was multiplied by the slice thickness to yield the fat volume (Fig. S1). Total EFV and PFV were obtained after summation of the corresponding data of all slices [13]. Indexed measures of the epi- and pericardial fat volumes were determined. Epicardial fat mass was determined by multiplying the measured EFV by the specific density of fat (0.92 g/cm3) [14]. Intra- and inter-observer variability for EFV and PFV was assessed among a random sample of 15 patients.

The study was approved by our institutional ethical committee (IRB:17101071). Patients provided informed consent for participation.

### Statistical analysis

Statistical analysis was performed using IBM-SPSS-24. Continuous variables were presented as mean ± standard deviation, and median (interquartile range) according to data distribution. Categorical variables were presented as frequencies (percentages). Quantitative variables were compared using Student t-test for normally distributed data, and Mann-Whitney-U for non-normally distributed data. One-way ANOVA was used to compare data among diabetic subgroups. Categorical data was compared using Chi^2^-test. Correlation between variables was done using Pearson`s test. Receiver Operating Characteristic Curve (ROC) analysis was used to determine the best cut-off values of EFV and PFV to predict severe CAD “high-SS”. Logistic regression analysis was performed to investigate predictors of high-SS. Reliability analysis was performed using intra-class correlation coefficient. *p* < 0.05 indicated statistical significance.

## Results

The study included 111 patients, of whom 64 (57.7%) were diabetic. The diabetic group had significantly older age, more hypertension and dyslipidemia, larger BMI, higher AIP, and higher SS compared to the non-diabetic group (Table 1). Results showed that only 18.8% of the diabetic patients achieved tight-control target (HbA1c < 7.0%), 31.3% had HbA1c in the range 7.0-8.4% (relax-control), and 50% were uncontrolled (HbA1c ≥ 8.5%). Comparative analysis among the three sub-groups (Table S1) showed that those with uncontrolled DM had significantly lower HDL and higher SS. They also had larger epicardial and pericardial fat volumes compared to the controlled ones, yet the difference was not statistically significant.

**Table 1:** Demographic and clinical data of the study population

	All individuals (*n* = 111)	Diabetics (*n* = 64) (57.7%)	Non-diabetics (*n* = 47) (42.3%)	*P* value
Age (years)	57.23 ± 11.58	60.81 ± 8.56	52.34 ± 13.34	< 0.001
Male Gender	102(85.7%)	53(82.8%)	42(89.4%)	0.33
Smoking	97(81.5%)	53(82.8%)	37(78.7%)	0.58
Hypertension	57(47.9%)	39(60.9%)	12(25.5%)	< 0.001
Dyslipidemia	27(22.7%)	19(29.7%)	5(10.6%)	0.01
BMI (kg/m^2^)	27.47 ± 4.72	28.31 ± 4.65	26.34 ± 4.62	0.02
BSA (m^2^)	1.83 ± 0.18	1.86 ± 0.17	1.80 ± 0.19	0.06
Lipid profile				
Total cholesterol (mg/dl)	206.96 ± 65.85	219.52 ± 69.28	189.77 ± 57.19	0.01
Triglycerides (mg/dl)	237.03 ± 101.87	254.97 ± 90.72	212.45 ± 111.81	0.03
HDL-C (mg/dl)	33.22 ± 6.37	33.14 ± 6.74	33.34 ± 5.89	0.87
LDL-C (mg/dl)	132.54 ± 42.64	140.40 ± 45.06	121.78 ± 36.88	0.02
Triglycerides/HDL ratio	7.54 ± 3.95	8.26 ± 3.98	6.56 ± 3.74	0.02
Atherogenic plasma index	0.81 ± 0.24	0.86 ± 0.23	0.75 ± 0.24	0.01
HbA1c	7.28 ± 1.72	8.41 ± 1.43	5.75 ± 0.35	< 0.001
Tight-Control (6.5–6.9%)	–	12(18.8%)	–	–
Relax-Control (7.0-8.4%)	–	20(31.3%)	–	–
Coronary angiography data				
LM disease	18(16.2%)	14(21.9%)	4(8.5%)	0.05
Single-vessel disease	39(35.1%)	13(20.3%)	26(55.3%)	< 0.001
Two-vessel disease	23(20.7%)	14(21.9%)	9(19.1%)	0.72
Three-vessel disease	49(44.1%)	37(57.8%)	12(25.5%)	0.001
Syntax Score	28.62 ± 14.70	33.57 ± 13.56	21.88 ± 13.56	< 0.001
Low SS (≤ 22)	41(36.9%)	16(25.0%)	25(53.2%)	0.002
Intermediate SS (> 22–32)	22(19.8%)	12(18.8%)	10(21.3%)	0.74
High SS (> 32)	48(43.2%)	36(56.3%)	12(25.5%)	0.001

*BMI* body mass index, *BSA* body surface area, *HbA1c* glycated hemoglobin, *HDL* high density lipoprotein, *LDL* low density lipoprotein, *LM* left main coronary artery, *SS* syntax score

CMR data (Table 2) showed that both study groups had comparable LV volumes and EF and comparable proportion of non-viable coronary territories. On the other hand, absolute and indexed EFV and PFV, as well as the total fat volumes were significantly higher among the diabetic group. Notably, both groups had comparable epicardial-to-pericardial fat ratio. Intra-class correlation coefficients of intra-observer variability for EFV and PFV were 0.96 and 0.95, respectively, and those for inter-observer variability were 0.96 and 0.89, respectively, (*p* < 0.001 for all).

**Table 2:** CMR data of the study population

	All individuals (*n* = 111)	Diabetics (*n* = 64) (57.7%)	Non-diabetics (*n* = 47) (42.3%)	*P* value
LA volume (ml)	91.77 ± 28.35	90.53 ± 24.70	93.59 ± 33.22	0.59
LV EDV (ml)	209.18 ± 68.96	205.14 ± 63.57	214.69 ± 76.06	0.47
LV ESV (ml)	137.32 ± 65.31	135.43 ± 58.24	139.91 ± 74.44	0.72
LV ejection fraction (%)	37.09 ± 11.94	35.90 ± 10.46	38.71 ± 13.65	0.22
LV ejection fraction ≤ 40%	70(63.1%)	44(68.8%)	26(55.3%)	0.14
Stroke volume (ml)	71.90 ± 16.76	69.67 ± 16.84	74.94 ± 16.35	0.10
Cardiac output (L/min)	5.41 ± 1.30	5.31 ± 1.38	5.56 ± 1.19	0.31
ED wall mass (gm)	127.15 ± 38.54	129.55 ± 39.02	123.70 ± 38.04	0.45
Non-viable LAD territory	23(20.7%)	12(18.8%)	11(23.4%)	0.55
Non-viable LCX territory	5(4.5%)	3(4.7%)	2(4.3%)	0.91
Non-viable RCA territory	4(3.6%)	4(6.3%)	0(0.0%)	0.08
Epicardial fat volume (ml)	109.48 ± 33.18	122.69 ± 30.73	91.49 ± 27.66	< 0.001
EFV indexed (ml/m^2^)	59.86 ± 18.27	66.02 ± 16.89	51.48 ± 16.83	< 0.001
Epicardial fat mass (gm)	100.72 ± 30.52	112.87 ± 28.27	84.17 ± 25.45	< 0.001
Pericardial fat volume (ml)	112.53 ± 35.82	125.94 ± 32.85	94.27 ± 31.61	< 0.001
PFV indexed (ml/m^2^)	61.53 ± 19.52	67.63 ± 17.17	53.22 ± 19.61	< 0.001
Total EFV and PFV (ml)	222.02 ± 66.06	248.63 ± 60.33	185.77 ± 55.87	< 0.001
Ratio EFV/PFV	0.99 ± 0.18	0.99 ± 0.15	0.99 ± 0.21	0.80
CA/CMR difference days	60(25–77)	60.5(30–74)	53(21–84)	0.83

*CA* coronary angiography, *CMR* cardiac magnetic resonance, *EDV* end-diastolic volume, *EFV* epicardial fat volume, *ESV* end-systolic volume, *LA* left atrium, *LAD* left anterior descending, *LCX* left circumflex, *LV* left ventricle, *PFV* pericardial fat volume, *RCA* right coronary artery

**Fig. 1:**
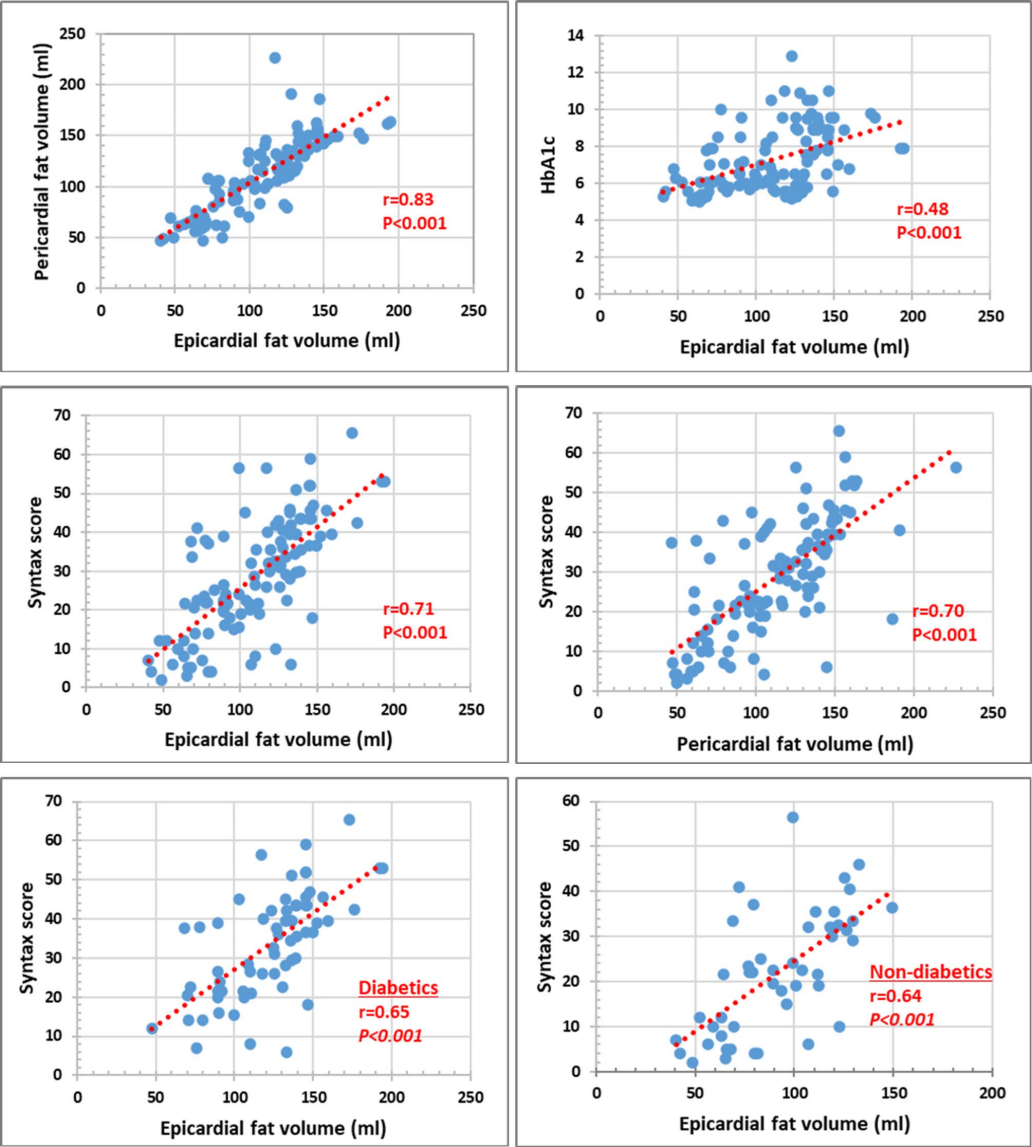
Correlations between Epicardial and pericardial fat volumes, HbA1c and Syntax score among the whole study population (upper and middle panels), and correlation between epicardial fat volume and syntax score among diabetics and non-diabetics (lower panel). *r* (correlation coefficient) and *p* (significance of correlation)

Our results showed that both EFV and PFV were strongly correlated (*r* = 0.83, *p* < 0.001). Moreover, both EFV and PFV showed significant moderate correlation with HbA1c (*r* = 0.48 and *r* = 0.47, respectively, *p* < 0.001) and strong correlation with the SS (*r* = 0.71 and *r* = 0.70, respectively, *p* < 0.001) among the whole study population. Further analysis showed that EFV maintained a significant moderate-strong positive correlation with the SS among both diabetics and non-diabetics (Fig. 1). On the other hand, both EFV and PFV showed weak negative correlation with LV EF (*r*=-0.30, *p* = 0.001) and (*r*=-0.27. *p* = 0.004), respectively. Noticeably, results showed that EFV and PFV were significantly larger among patients with LV EF ≤ 40% compared to those with LV EF > 40% (116.2 ± 33.3 vs. 98.0 ± 30.0, *p* = 0.005) and (118.7 ± 34.2 vs. 102.0 ± 36.5, *p* = 0.01), respectively. Moreover, univariable logistic regression analysis showed that increased EFV significantly predicted impaired LV EF ≤ 40% (OR = 0.98, CI = 0.97–0.99, *p* = 0.007), and similarly were the results for increased PFV (OR = 0.98, CI = 0.97–0.99, *p* = 0.01).

**Fig. 2:**
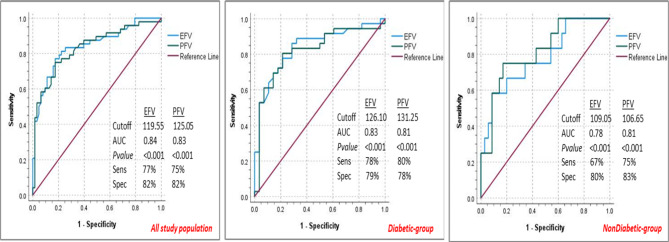
Receiver-operating characteristic (ROC) analysis for the predictability of epicardial and pericardial fat volumes for high syntax among the whole population (left), diabetics (middle), and non-diabetics (right). *AUC* area under the curve, *EFV* epicardial fat volume, *PFV* pericardial fat volume

Angiographic data showed that 43.2% had high SS. The high-SS group demonstrated significantly higher HbA1c, lower LV EF, higher rates of non-viable RCA-territory, and significantly larger both EFV and PFV compared to the low/intermediate-SS (Table S2). ROC-analysis performed to assess the predictability of epi- and pericardial fat volumes for severe CAD among the study population (Fig. 2) showed that EFV ≥ 119.55 ml (AUC = 0.84, 95%CI = 0.76–0.91, sensitivity = 77% and specificity = 82.5%) and PFV ≥ 125.05 ml (AUC = 0.83, 95%CI = 0.75–0.91, sensitivity = 75% and specificity = 82.5%) significantly predicted high-SS. Sub-analysis performed for the diabetic and non-diabetic groups independently showed significant predictability of both epi- and pericardial fat volumes for high SS at different cutoffs with respective reasonable sensitivity and specificity.

Univariable regression analysis (Table 3) showed that older age, hypertension, dyslipidemia, elevated triglycerides, elevated LDL, lowered HDL, high AIP, high HbA1c, as well as high absolute and indexed EFV and PFV were significantly associated with severe and complex CAD as indicated by high SS. Noticeably, uncontrolled diabetes with HbA1c ≥ 8.5% showed higher odds for increased SS than HbA1c ≥ 7.0% (7.45 vs. 4.66). Adjusted for other covariates, age, and EFV were significant independent predictors of high SS on different multivariable regression models.

**Table 3:** Predictors of severe and complex CAD (high syntax score)

Variables	Univariable analysis			Multivariable analysis		
	OR	95% CI	*P* value	OR	95% CI	*P* value
*Logistic regression analysis for predictors of high syntax score among the whole study population*						
Age	1.12	1.06–1.17	< 0.001	^(1)^1.09	1.03–1.16	0.002
				^(2)^1.11	1.04–1.19	0.002
				^(3)^1.12	1.05–1.21	0.001
Hypertension	2.43	1.12–5.25	0.02	^(1)^ 1.19	0.39–3.62	0.75
				^(2)^ 0.71	0.21–2.42	0.59
				^(3)^ 0.68	0.20–2.33	0.54
Dyslipidemia	2.72	1.07–6.93	0.03	^(1)^ 1.05	0.31–3.49	0.93
Total cholesterol (mg/dl)	1.00	0.99–1.01	0.11			
Triglycerides (mg/dl)	1.00	1.00-1.01	0.08			
LDL-C (mg/dl)	1.01	1.00-1.02	0.001	^(2)^ 1.01	0.99–1.03	0.09
				^(3)^ 1.02	1.00-1.03	0.05
HDL-C (mg/dl)	0.87	0.80–0.94	0.001			
Triglycerides/HDL ratio	1.15	1.03–1.27	0.009			
Atherogenic Plasma Index	13.94	2.32–83.77	0.004	^(2)^ 2.14	0.08–54.10	0.64
				^(3)^ 0.99	0.04–24.83	0.99
HbA1c (%)	1.70	1.31–2.21	< 0.001			
Diabetes (HbA1c ≥ 6.5%)	3.75	1.65–8.52	0.002	^(1)^ 1.06	0.33–3.45	0.91
HbA1c ≥ 7.0%	4.66	1.97–11.04	< 0.001			
HbA1c ≥ 8.5%	7.45	2.71–20.50	< 0.001	^(2)^ 1.76	0.40–7.79	0.45
				^(3)^ 2.36	0.54–10.31	0.25
LV ejection fraction (%)	0.94	0.91–0.98	0.003	^(1)^ 0.96	0.90–1.01	0.13
				^(2)^ 0.95	0.91–1.01	0.11
				^(3)^ 0.95	0.90–1.01	0.10
Epicardial fat volume (ml)	1.05	1.03–1.07	< 0.001	^(1)^1.04		
				^(2)^1.03	1.01–1.06 1.01–1.05	< 0.001 0.003
EFV indexed (ml/m^2^)	1.09	1.05–1.13	< 0.001			
EFV ≥ 119.55 ml	15.90	6.23–40.54	< 0.001	^(3)^7.02	2.19–22.49	0.001
Epicardial fat mass	1.05	1.03–1.07	< 0.001			
Pericardial fat volume (ml) #	1.04	1.02–1.06	< 0.001			
PFV indexed (ml/m^2^)	1.07	1.04–1.11	< 0.001			
Total EFV and PFV (ml)	1.02	1.01–1.03	< 0.001			

*CI* confidence interval, *EFV* epicardial fat volume, *HbA1c* glycated hemoglobin, *HDL* high density lipoprotein, *LDL* low density lipoprotein, *LV* left ventricle, *OR* Odds ratio, *PFV* pericardial fat volume

^(1)^Model 1: age, hypertension, dyslipidemia, diabetes, LV EF, and EFV

^(2)^Model 2: age, hypertension, LDL, atherogenic plasma index, HbA1c ≥ 8.5%, LV EF, and EFV

^(3)^Model 3: age, hypertension, LDL, atherogenic plasma index, HbA1c ≥ 8.5%, LV EF, and EFV ≥ 119.55 ml

# EFV and PFV were not included together in multivariable regression models to avoid multicollinearity

## Discussion

The main findings of our study showed that EFV and PFV were significantly increased among diabetic patients, who in turn had significantly higher SS and multivessel CAD. Both EFV ≥ 119.55 ml and PFV ≥ 125.05 ml showed significantly strong predictability for high-SS. This was consistently found at different cutoffs among diabetics and non-diabetics with respective reasonable sensitivity and specificity. Furthermore, on multivariable regression analysis models corrected for HbA1c level, and other covariables, increased EFV was a significant independent predictor of high-SS along with age.

CMR provides an ideal gold standard for accurately measuring true epi- and pericardial fat volumes. Multiple CMR-based studies have demonstrated significantly larger EAT among diabetic patients in a variety of clinical settings [15], however with deficient data regarding its relation to CAD complexity. To our knowledge, this is the first study to comprehensively assess both EFV and PFV, as well as their impact on CAD severity and complexity among diabetics versus non-diabetics.

### Association between epicardial adipose tissue and severity of CAD among diabetics

Epicardial adipose tissue is an active endocrine organ that secretes different adipocytokines via endocrine and paracrine routes to exert various cardiovascular effects. It was linked not only to CAD progression but also cardiomyopathy, particularly in diabetics, arrhythmias e.g. AF, and other cardiovascular and metabolic abnormalities [16]. The relation between EAT and the heart is bidirectional, as EAT exerts paracrine effects on the latter (outside-in) but is also influenced by inflammatory mediators generated in the vascular wall (inside-out), causing changes in the secreted adipokines [17].

Results of our study demonstrated significantly larger EFV and PFV in association with both diabetes and increased complexity of CAD. It was previously demonstrated that both diabetes and increased epicardial fat volume mutually co-exist and synergistically enhance atherosclerosis [2]. This was evident among our study population; whereby diabetic patients had significantly larger EFV, larger BMI, altered plasma cholesterol levels with significantly higher AIP, in association with significantly higher rates of multivessel CAD and higher SS. Presence of either diabetes or increased EFV was demonstrated to interchangeably enhance the development of the other, inducing a state of insulin resistance, systemic inflammation, and excess secretion of proinflammatory adipokines with deleterious effects, which explain the potential biological mechanisms beyond their role in the development of CAD with further increased severity and complexity [3, 4].

Our findings were consistent with the results of a meta-analysis of 21 studies (including 2377 patients with variable degrees of CAD and 2598 participants with no CAD), that showed significantly larger EAT (either echo-measured thickness or CT-measured volume) among the CAD group, and in turn among those having significant stenosis (≥ 50%), however, it did not report about differences in relation to DM and was limited by marked heterogeneity of the included studies [18]. Another meta-analysis including 13 studies (11 echo-measured thickness and 2 CT-measured EFV) with 1102 diabetic patients, demonstrated significantly increased EAT among diabetics compared to control [19].

A previous CMR study demonstrated that increased epicardial fat thickness was significantly associated with a high risk of the composite outcome of myocardial infarction, ischemic stroke, heart failure, and cardiac death among 1554 participants over a median follow-up of 12.7years [20]. On the other hand, it has been suggested that treatment with new antidiabetic drugs as SGLT2 inhibitors [21] and GLP-1 analogues [22] reduced cardiovascular risk and mortality possibly induced by the associated reduction in EAT.

Contradictory results were presented by vanMeijeren et al., demonstrating that CMR-measured EFV was not independently associated with stages of CAD [13]. However, limited by the relatively small sample of CAD patients, the severity of atherosclerosis was not assessed, strict inclusion criteria (excluding diabetics and BMI > 35Kg/m^2^), and partial confounding due to use of intense statin therapy reflecting inclusion of an already high cardiovascular risk population.

The advantages of CMR extend beyond the mere precise assessment of true epi- and pericardial fat volumes to the exploration of the structural and functional consequences of increased fat volume on adjacent myocardial tissue with possible fatty infiltration. This might provide better demonstration of the impact of increased EFV among diabetics in association with complex CAD. In this respect, results of a recently published study by Bialobroda et al. demonstrated that diabetic patients had significantly increased EFV measured at the atrio-ventricular groove and altered epicardial fat structure evident by decreased T1 relaxation times, that was associated with decreased atrial strain reflecting impaired atrial myocardial function [23]. In our study, myocardial fibrosis was detected grossly using the phase-sensitive inversion recovery technique on late gadolinium enhancement images for assessment of viability based on the percentage of subendocardial enhancement. Unfortunately, our study was limited to the volumetric fat assessment due to unavailability of the respective software analysis tools for T1 and T2 mapping sequences.

On the other hand, previous studies demonstrated an association between EFV and heart failure, whereby increased EFV had deleterious effects in patients having HF with mid-range and preserved EF [24], yet paradoxically improved LV structural and functional consequences among patients with dilated cardiomyopathy [25]. Our study comprised an entity of patients with established CAD who presented for viability assessment, expecting that most of them had severe LV dysfunction secondary to significant CAD. The interplay of diabetes associated with increased inflammatory state, increased EFV, and presence of significant CAD might have impacted the degree of LV dysfunction. Our results demonstrated comparably low LV EF among diabetics and non-diabetics, yet significantly lower LV EF among patients with high SS compared to the low/intermediate SS. On the other hand, EFV showed weak negative correlation with LV EF, yet significantly predicted impaired LV EF ≤ 40%. This was consistent with previous data showing that increased EFV was implicated in the development of obstructive CAD as well as in non-ischemic diabetic cardiomyopathy [15]. Moreover, studies demonstrated that using SGLT2i was associated with reduced EFV and improved LV function regardless of the presence or absence of diabetes [26–28].

### Impact of the glycemic state on measured epicardial and pericardial fat volumes

Furthermore, results of our study demonstrated that on sub-analysis of the diabetic group, both EFV and PFV tended to have a descending pattern of smaller volumes in relation to the glycemic-control categories (uncontrolled (HbA1c ≥ 8.5%), relax-control (HbA1c 7.0-8.4%), and tight-control (HbA1c 6.5–6.9%), respectively), however with borderline statistical significance. Our relatively small sample size might have limited achievement of evident conclusion in this regard. To our knowledge, there is no available data in the literature regarding the impact of the degree of glycemic control on cardiac adipose tissue volume. However, it was previously demonstrated that weight-reduction interventions such as diet, exercise, pharmacological interventions (including antidiabetic drugs), and bariatric surgery significantly reduced EFV [29, 30].

In contrary, Iacobellis et al. demonstrated that echo-measured epicardial fat thickness predicted the development of CAD at 1-year follow-up among well-controlled asymptomatic diabetic obese patients with baseline HbA1c = 6.7% and BMI = 34.9Kg/m^2^ better than other traditional risk factors including age, hypertension, BMI, and duration of DM [31]. Thus, eliminating the effect of the glycemic state in favor of epicardial fat thickness.

### Predictability of EFV for severity of CAD among diabetics

Results of our study demonstrated a significant strong positive correlation between each of EFV and PFV and the syntax score, and significant association of each with high SS. Moreover, our results showed that adjusted for other covariates (including elevated LDL, high atherogenic plasma index, elevated HbA1c, and lowered LV EF), age, and EFV independently predicted high SS on different multivariable regression models.

Previous data showed that echo-measured epicardial fat thickness was positively associated with increased severity of CAD assessed by the Gensini score among diabetic patients [32]. Few CT-volumetric studies demonstrated a significant association of EFV with significant CAD but did not report on their association among diabetics versus non-diabetics. It was shown that CT-measured EFV significantly correlated with and independently predicted the presence and severity of hemodynamically significant CAD that was automatically assessed using quantitative flow ratio [33]. Yu et al., demonstrated that CT-measured EFV ≥ 134.43cm^3^ was associated with hemodynamically significant CAD (≥ 50% luminal stenosis and with reversible corresponding perfusion defects on single-photon emission computerized tomography-myocardial perfusion imaging (SPECT/MPI) [34]. Moreover, increased EFV was predictive of major adverse cardiovascular events with a follow-up of 76 months [35].

Importantly, our study demonstrated strong predictability of CMR-measured EFV and PFV cutoff values for high-SS among the whole study population (119.55 ml and 125.05 ml, respectively) as well as among both diabetics (126.10 ml and 131.25 ml, respectively) and non-diabetics (109.05 ml and 106.65 ml, respectively). Presenting these cutoffs as strong predictors for increased complexity of CAD is more clinically meaningful than the mere unit increase in volume. This underscores the importance of testing these proposed thresholds among larger studies or developing new ones to be incorporated among traditional risk scores and to be used as therapeutic targets.

### Possible role of pericardial adipose tissue in coronary atherosclerosis

Despite the embryological, anatomical, and functional differences between epicardial and pericardial adipose tissues, the latter may be indirectly implicated in the process of atherosclerosis being involved in the chronic inflammatory state seen among diabetics. Few studies showed that a combined increase in both would impact CAD risk and outcomes, particularly among diabetics, yet the underlying mechanism remains unclear [30, 36]. Echo-measured total cardiac adipose tissue above the median value (8.75 mm) was shown to be associated with high cardiovascular risk and all-cause mortality after a follow-up of 6 years among 200 diabetic patients [37]. A previous CT study that assessed pericardial fat enhancement, as a marker of inflammation, demonstrated a significant independent association between PFV and obstructive CAD (OR = 1.26, *p* = 0.005), as well as CAD burden (OR = 1.25, *p* = 0.05) in those with greater pericardial fat enhancement [38]. In this respect, our study demonstrated significantly higher levels of EFV, PFV, and their sum among diabetics versus non-diabetics. However, the ratio of epicardial to pericardial fat volumes was comparable among the study groups, denoting maintained relation with possible interaction of both in the pathogenesis of CAD. Moreover, our study demonstrated that increased PFV was significantly associated with higher odds of severe CAD (high-SS) and proposed respective cutoff values among the general population and selectively among diabetics as well as non-diabetics. To our knowledge, this was not assessed in other comparative CMR-volumetric studies, necessitating further investigation.

### Limitations

The cross-sectional study design and the relatively small sample size might have limited our results. Our study focused on CMR-measured epi- and pericardial fat volumes, however, did not assess their corresponding echo- and CMR-measured thickness. This might have limited the ability to translate our findings into a simple, easily accessible risk assessment tool for daily clinical practice, albeit provided reliable precise assessment of both epi- and pericardial fat volumes with proposed cutoff thresholds in association with severe CAD among the general population and selectively among diabetics and non-diabetics.

## Conclusions

Our study demonstrated a significant association between CMR-measured epicardial, and pericardial fat volumes and the complexity of CAD among diabetics as well as non-diabetics. At a cutoff value of 119.55 ml, EFV significantly predicted high-SS. Adjusting for other covariables, age and higher EFV were independent predictors of complex CAD.

Further studies assessing the integration of EAT thickness/volume in risk models, defining cutoff thresholds or testing our proposed ones, as well as evaluating its potential as a therapeutic target particularly among diabetics warrant assessment.

## Electronic supplementary material

Below is the link to the electronic supplementary material.

Supplementary Table S1. Comparative data among diabetic subgroups according to HbA1c level. Table S2. Comparative data between low/intermediate and high syntax score. Figure S1. Example of CMR-measured epicardial and pericardial fat volumes.

## Data Availability

No datasets were generated or analysed during the current study.
