# Supplementary material for: Cardiac magnetic resonance quantified epicardial fat volume is associated with complex coronary artery disease among diabetics
Source: Cardiovasc Diabetol. 2025 Feb 7;24:64. doi: 10.1186/s12933-025-02606-x (PMC11806836; doi:10.1186/s12933-025-02606-x)
Supplement: Supplementary file 1 — Supplementary Table S1. Comparative data among diabetic subgroups according to HbA1c level. Table S2. Comparative data between low/intermediate and high syntax score. Figure S1. Example of CMR-measured epicardial and pericardial fat volumes. [file 12933_2025_2606_MOESM1_ESM.docx]

**Table S1. Comparative data among diabetic subgroups according to HbA1c level**

|  | **Tight-Control**  **(HbA1c 6.5-6.9%)**  **(n=12) (18.8%)** | **Relax-Control**  **(HbA1c 7.0-8.4%)**  **(n=20) (31.3%)** | **Uncontrolled**  **(HbA1c≥8.5%)**  **(n=32) (50.0%)** | ***P* value** |
| --- | --- | --- | --- | --- |
| **Age** (years) | 58.50±9.77 | 60.90±6.35 | 61.63±9.63 | 0.56 |
| **Male Gender** | 10 (83.3%) | 16(80.0%) | 27(84.4%) | 0.91 |
| **Smoking** | 9 (75.0%) | 16(80.0%) | 28(87.5%) | 0.57 |
| **Hypertension** | 3(25.0%) | 14(70.0%) | 22(68.8%) | **0.01** |
| **Dyslipidemia** | 3(25.0%) | 5(25.0%) | 11(34.4%) | 0.71 |
| **BMI** (kg/m^2^) | 27.00±4.10 | 27.93±3.06 | 29.03±5.56 | 0.40 |
| **BSA** (m^2^) | 1.83±0.13 | 1.84±0.15 | 1.86±0.17 | 0.58 |
| **Lipid profile**  Total cholesterol (mg/dl)  Triglycerides (mg/dl)  HDL-C (mg/dl)  LDL-C (mg/dl)  Triglycerides/HDL ratio  Atherogenic Plasma Index | 205.69±49.92  231.58±84.92  36.71±8.11  135.32±46.34  6.89±3.52  0.78±0.24 | 209.56±60.00  241.22±78.95  34.95±6.76  132.37±40.48  7.40±3.51  0.82±0.22 | 230.62±79.77  271.91±98.27  30.72±5.29  147.06±47.46  9.29±4.22  0.91±0.22 | 0.43  0.31  **0.01 #**  0.49  0.10  0.13 |
| **HbA1c** (%) *** # ˟** | 6.60±0.14 | 7.60±0.42 | 9.60±0.95 | **<0.001** |
| LM disease  Single-vessel disease  Two-vessel disease  Three-vessel disease | 2(16.7%)  4(33.3%)  2(16.7%)  6(50.0%) | 3(15.0%)  6(30.0%)  6(30.0%)  8(40.0%) | 9(28.1%)  3(9.4%)  6(18.8%)  23(71.9%) | 0.47  0.09  0.56  0.06 |
| **Syntax Score**  Low SS (≤22)  Intermediate SS (>22-32)  High SS (>32) | 26.20±9.09  5(41.7%)  3(25.0%)  4(33.3%) | 31.70±14.32  7(35.0%)  4(20.0%)  9(45.0%) | 37.50±13.41  4(12.5%)  5(15.6%)  23(71.9%) | **0.03 #**  0.06  0.76  **0.03** |
| **LV EDV** (ml) | 204.95±67.66 | 199.08±56.51 | 209.00±67.76 | 0.86 |
| **LV ESV** (ml) | 134.29±61.92 | 131.06±49.52 | 138.58±63.31 | 0.90 |
| **LV ejection fraction** (%) | 36.60±9.89 | 35.29±10.96 | 36.03±10.67 | 0.94 |
| **LV ejection fraction ≤40%** | 8(66.7%) | 13(65.0%) | 23(71.9%) | 0.49 |
| **Non-viable LAD territory**  **Non-viable LCX territory**  **Non-viable RCA territory** | 1 (8.3%)  1 (8.3%)  0(0.0%) | 5(25.0%)  0(0.0%)  1(5.0%) | 6(18.8%)  2(6.3%)  3(9.4%) | 0.50  0.46  0.50 |
| **Epicardial fat volume** (ml) | 109.36±29.70 | 118.00±38.42 | 132.62±23.54 | 0.08 |
| **Pericardial fat volume** (ml) | 112.66±26.57 | 118.90±36.63 | 135.32±30.49 | 0.06 |
| **Total EFV and PFV** (ml) | 222.03±43.89 | 236.90±72.99 | 265.95±49.29 | 0.05 |

**BMI:** body mass index, **BSA:** body surface area, **EDV:** end-diastolic volume, **EFV:** epicardial fat volume, **ESV:** end-systolic volume, **HbA1c:** glycated hemoglobin, **HDL**: high density lipoprotein, **LAD**: left anterior descending, **LCX**: left circumflex, **LDL**: low density lipoprotein, **LM:** left main coronary artery, **LV:** left ventricle, **PFV:** pericardial fat volume, **RCA:** right coronary artery, **SS:** syntax score.

***P*-value denotes between groups level of significance. *#˟ denote presence of within group significant difference between tight-control and relax-control, tight-control and uncontrolled, and relax-control and uncontrolled subgroups, respectively.**

**Table S2. Comparative data between low/intermediate and high syntax score**

|  | **Low/Intermediate SS**  **(n=63) (56.8%)** | **High SS**  **(n=48) (43.2%)** | ***P* value** |
| --- | --- | --- | --- |
| **Age** (years) | 52.52±11.81 | 63.40±7.83 | **<0.001** |
| **Male Gender** | 53(84.1%) | 42(87.5%) | 0.61 |
| **Smoking** | 48(76.2%) | 42(87.5%) | 0.13 |
| **Hypertension** | 23(36.5%) | 28(58.3%) | **0.02** |
| **Dyslipidemia** | 9(14.3%) | 15(31.3%) | **0.03** |
| **BMI** (kg/m^2^) | 27.43±4.56 | 27.53±4.97 | 0.91 |
| **BSA** (m^2^) | 1.83±0.19 | 1.83±0.16 | 0.94 |
| **Lipid profile**  Total cholesterol (mg/dl)  Triglycerides (mg/dl)  HDL-C (mg/dl)  LDL-C (mg/dl)  Triglycerides/HDL ratio  Atherogenic Plasma Index | 198.33±59.46  222.05±104.99  35.11±6.19  120.13±37.30  6.65±3.76  0.75±0.24 | 218.35±72.51  256.86±95.09  30.73±5.78  148.91±44.06  8.72±3.93  0.89±0.20 | 0.11  0.07  **<0.001**  **<0.001**  **0.006**  **0.003** |
| **HbA1c** | 6.68±1.39 | 8.08±1.80 | **<0.001** |
| **Diabetics**  *Tight-Control (6.5-6.9%)  *Relax-Control (7.0-8.4%)  *Uncontrolled (≥8.5%) | 28(44.4%)  8/28(28.6%)  11/28(39.3%)  9/28(32.1%) | 36(75.0%)  4/36(11.1%)  9/36(25.0%)  23/36(63.9%) | **0.001**  **0.03** |
| **LA volume** (ml) | 89.85±30.18 | 94.16±20.03 | 0.45 |
| **LV EDV** (ml) | 199.73±66.80 | 221.59±70.49 | 0.09 |
| **LV ESV** (ml) | 125.70±63.37 | 152.58±65.31 | **0.03** |
| **LV ejection fraction** (%) | 40.15±12.72 | 33.08±9.56 | **0.002** |
| **LV ejection fraction** ≤40% | 34(54.0%) | 36(75.0%) | **0.02** |
| **Non-viable LAD territory**  **Non-viable LCX territory**  **Non-viable RCA territory** | 11(17.5%)  2 (3.2%)  0(0.0%) | 12(25.0%)  3(6.3%)  4(8.3%) | 0.33  0.43  **0.02** |
| **Epicardial fat volume** (ml) | 92.97±26.52 | 131.16±28.34 | **<0.001** |
| **EFV indexed** (ml/m^2^) | 50.88±14.60 | 71.65±15.82 | **<0.001** |
| **Epicardial fat mass** | 85.53±24.39 | 120.66±26.07 | **<0.001** |
| **Pericardial fat volume** (ml) | 95.90±29.70 | 134.37±31.33 | **<0.001** |
| **PFV indexed** (ml/m^2^) | 52.58±16.76 | 73.28±16.52 | **<0.001** |
| **Total EFV and PFV** (ml) | 188.87±53.91 | 265.53±54.52 | **<0.001** |
| **Ratio EFV/PFV** | 0.99±0.18 | 0.99±0.17 | 0.82 |

**BMI:** body mass index, **BSA:** body surface area, **HbA1c:** glycated hemoglobin, **HDL**: high density lipoprotein, **LA:** left atrium, **LDL**: low density lipoprotein, **LM:** left main coronary artery.

***Displayed proportions are for diabetic subgroups among the corresponding syntax categories.**


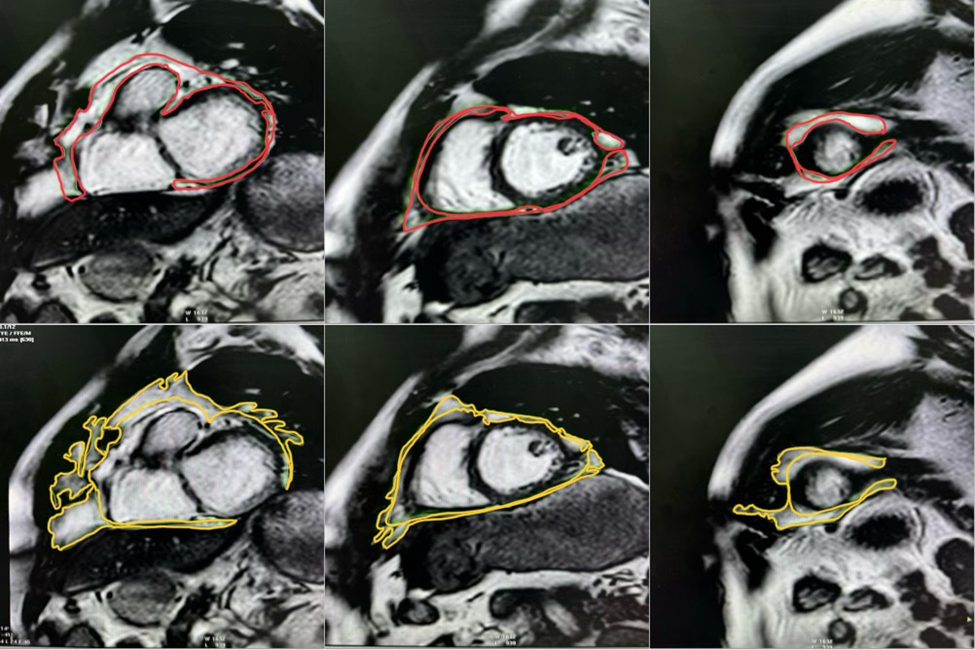


**Figure S1. Example of CMR-measured epicardial and pericardial fat volumes among a diabetic patient.** Epicardial fat tracing (upper panel) and pericardial fat tracing (lower panel) at the basal, mid, and apical short-axis images, respectively. Male patient 54 years, known to be diabetic (HbA1c=9.6%), hypertensive and has chronic stable angina. Coronary angiography showed multi-vessel disease and high syntax score of 47. CMR-derived EFV=148ml and PFV=146.2ml. Both EFV and PFV were significantly enlarged beyond the cutoffs demonstrated by our study to predict high-SS.
